# Video-based, student tutor- versus faculty staff-led ultrasound course for medical students – a prospective randomized study

**DOI:** 10.1186/s12909-020-02431-8

**Published:** 2020-12-16

**Authors:** Christine Eimer, Max Duschek, Andreas Emanuel Jung, Günther Zick, Amke Caliebe, Matthias Lindner, Norbert Weiler, Gunnar Elke

**Affiliations:** 1 Department of Anaesthesiology and Intensive Care Medicine, University Medical Center Schleswig-Holstein, Campus Kiel, Arnold-Heller-Str. 3 Haus R3, 24105 Kiel, Germany; 2 Institute of Medical Informatics and Statistics, Christian-Albrechts-University Kiel, University Medical Center Schleswig-Holstein, Campus Kiel, 24105 Kiel, Germany

**Keywords:** Ultrasound, Education, Teaching, Medical student, Video-tutorial, OSCE, Undergraduate, Peer-teaching, Student tutor, Peer-to-peer teaching

## Abstract

**Background:**

Ultrasound education is propagated already during medical school due to its diagnostic importance. Courses are usually supervised by experienced faculty staff (FS) with patient bedside examinations or students among each other but often overbooked due to limited FS availability. To overcome this barrier, use of teaching videos may be advantageous. Likewise, peer teaching concepts solely with trained student tutors have shown to be feasible and effective. The aim was to evaluate 1) objective learning outcomes of a combined video-based, student-tutor (ViST) as compared to a FS-led course without media support, 2) acceptance and subjective learning success of the videos.

**Methods:**

Two ultrasound teaching videos for basic and advanced abdominal ultrasound (AU) and transthoracic echocardiography (TTE) were produced and six students trained as tutors. Fourth-year medical students (*N* = 96) were randomized to either the ViST- or FS course (6 students per tutor). Learning objectives were defined equally for both courses. Acquired practical basic and advanced ultrasound skills were tested in an objective structured clinical examination (OSCE) using modified validated scoring sheets with a maximum total score of 40 points. Acceptance and subjective learning success of both videos were evaluated by questionnaires based on Kirkpatrick’s evaluation model with scale-rated closed and open questions.

**Results:**

79 of 96 medical students completed the OSCE and 77 could be finally analyzed. There was no significant difference in the mean total point score of 31.3 in the ViST (*N* = 42) and 32.7 in the FS course (*N* = 35, *P* = 0.31) or in any of the examined basic or advanced ultrasound skill subtasks. Of the 42 ViST participants, 29 completed the AU and 27 the TTE video questionnaire. Acceptance and subjective learning success of both videos was rated positively in 14–52% and 48–88% of the rated responses to each category, respectively. Attendance of either the student or faculty tutor was deemed necessary in addition to the videos.

**Conclusions:**

A ViST versus FS teaching concept was able to effectively teach undergraduate students in AU and TTE, albeit acceptance of the teaching videos alone was limited. However, the ViST concept has the potential to increase course availability and FS resource allocation.

**Supplementary Information:**

The online version contains supplementary material available at 10.1186/s12909-020-02431-8.

## Background

Interest in ultrasound education early during medical school has increased tremendously in recent years due to its pivotal role as a diagnostic tool in nearly all medical disciplines. Although ultrasound is widely available and used in clinical practice, shortcomings in knowledge and skills still exist among medical students and younger physicians and curricular designs across undergraduate medical education programs remain variable without adoption of standards and guidelines [1–3]. The World Federation of Ultrasound in Medicine and Biology (WFUMB) has formulated a consensus statement on how to integrate ultrasound teaching into the preclinical and clinical medical curricula as in their view “medical education methodology, particularly in anatomy, pathophysiology and physical examination is undergoing a paradigm shift based on the application of ultrasound technology that will likely fundamentally change on how medicine is taught and practiced” [4, 5]. Accordingly, they propagate the establishment of modern and novel systematic educational structures in a way that suits the specific educational needs of medical students and with the aim to increase understanding of anatomy, physiology and pathology early during medical education [4, 5].

Classically, theoretical ultrasound knowledge is taught teacher-centered in lectures or by the means of scripts followed by practical “hands-on” courses being held in small groups where students alternate in the role of examiner and patient under supervision of a physician or faculty staff member experienced in ultrasound [6, 7]. This practical ultrasound teaching with its student team-based approach sharing one ultrasound device allows to encompass collaborative learning as one variety of active learning. Collaborative learning is sought to enhance students’ active engagement and the quality of knowledge acquisition “where a group of learners works together in order to solve a problem or complete a task” [8, 9]. In a collaborative setting, learners are challenged not only academically but also socially and emotionally promoting teamwork competencies from an early stage on during medical education [10]. Still, the “classical” course concept is challenging mainly due to workload and personnel expenses of available clinical teachers particularly where faculty to student ratios are low [1, 11, 12].

An alternative way is peer-teaching as an educational format that recruits one or more students to acquire enough knowledge and skills to become teacher themselves helping fellow students to learn in a non-hierarchical, collaborative setting [13, 14]. Peer-teaching and reciprocally peer-assisted learning has the potential to improve both the student teacher and learner academic knowledge and interpersonal skills acquisition that can be applied in future clinical situations and is increasingly recognized as a core professional ability across all (health) professional disciplines [15, 16]. Peer-teaching has previously been shown to be as effective as faculty members to teach basic and even advanced point-of-care ultrasound (POCUS) including Extended Focused Assessment with Sonography for Trauma (eFAST), lung-ultrasound and Focused Echocardiography in Emergency Life Support (FEEL) [17–21]. The initiative “sono4students” has become the largest ultrasound peer-teaching platform in Germany [22].

Another contemporary way to increase the efficacy of undergraduate curricula is the integration of digital multimedia, social media or online learning applications [23–27]. A global online platform providing ultrasound teaching videos already exists [28]. These approaches for medical education have become even more important due to the ongoing COVID-19 pandemic [29]. Multimedia learning combines the use of both visual (i.e. static images such as illustrations or dynamic images such as animation) and verbal material (i.e. printed or spoken words) and is recommended to follow the research-based principles of the cognitive load and dual-channel coding theory for instructional design of multimedia lessons introduced by Mayer [30, 31]. Essentially, these 12 principles aim at reducing extraneous processing, managing essential processing and fostering generative processing when designing educational materials for medical students [32]. Implementation of the principles into medical classes has been shown to improve knowledge retention and transfer in undergraduate medical students [33, 34]. However, using teaching videos has shown divergent effects on learning outcomes and perception in different educational settings [25, 35–39]. Surprisingly, only two studies have yet evaluated the effect of video-based ultrasound teaching and showed that learning was effective but may not completely replace the need for supervision during practical exercises [40, 41].

Since 2015, our department has established a voluntary, extracurricular ultrasound course that constantly received positive feedback in our ongoing university student evaluation process and has been frequently overbooked due to faculty staff (FS) shortage. In order to overcome this barrier and increase course availability, we sought to combine the use of video and student peer teaching in a new and feasible curriculum concept for abdominal ultrasound (AU) and transthoracic echocardiography (TTE). The aim of our study was to analyze objective learning outcomes of this novel course concept as compared to the classical, faculty staff-led ultrasound course without media support. As a second objective, acceptance and subjective learning outcomes of the ultrasound teaching videos should be evaluated.

## Methods

### Study design and participants

This was a prospective randomized, single-blinded cohort study conducted by the Department of Anaesthesiology and Intensive Care Medicine, University Medical Center, Schleswig-Holstein, Campus Kiel/Christian-Albrechts-University, Kiel, Germany. Study participants were 96 medical students in their fourth year of medical school. The ethics committee of the Medical Faculty of the Christian-Albrechts-University Kiel, Germany confirmed that this study was not subject to consulting duty according to the professional medical code of conduct of the medical association Schleswig-Holstein (§ 15 BO) and the need for written informed consent was waived (file number of the ethics committee: D 554/19). All students were informed about the study procedures, anonymous data acquisition and that study participation was voluntary with the option to still participate in the FS-led course and not being included in the analysis in case of declining consent. All 96 study participants agreed to participate before the start of the study.

The students had no previous theoretical or practical knowledge in ultrasound and were allocated to both courses in random order (simple randomization using sealed envelopes) so that each course was carried out with 48 students. Both courses were conducted in parallel in small groups of six students per tutor and were correspondingly started with either the video tutorial or the live instruction of the faculty tutor. Then the practical course part was carried out where two of the six participants each shared an ultrasound device and were both ultrasound examiner and subject to be examined in an alternating fashion. Thus, active learning was included in the practical part of both courses (“skills-lab setting”) as this two-student team learned “hands-on” in a collaborative fashion. All participants attended two ultrasound sessions per course scheduled for two hours each, one session for AU and the other for TTE.

### Video-based, student tutor-led course

The video-based, student tutor-led (ViST) course included the presentation of the two educational videos for AU and TTE at the beginning followed by the small group practical exercises with supervision by the student peer-teacher. One ultrasound-experienced clinical teacher was present only as a back-up supervisor in case the student tutors required assistance. The AU video had a total length of 15 min, the TTE video 18 min. Participants watched each video as a class on an institutional computer for the first time in the respective AU or TTE course. Both videos were presented in total and - as per request – certain parts could be watched repeatedly with the possibility to rewind and at faster speed. No further opportunities were given to watch the videos again in order to have the same teaching time exposure as compared to the faculty staff-led course.

Prior to the course implementation, the concept had been developed in two parallel steps. First, six students were trained by two physicians with perennial experience in ultrasound in order to become ultrasound tutors themselves for the video-based course. Second, the two educational AU and TTE videos were specifically developed as part of a student project in cooperation with the Department of Multimedia Production, University of Applied Sciences, Kiel, Germany for graphical visualization and video-technical implementation support. In the design and production process of both videos, we followed 11 of the 12 principles of multimedia learning (with exclusion of the pre-training principle) [30, 31]. The main topic was to create a video tutorial showing the ultrasound exam step by step following the segmenting principle. Each video was segmented at the beginning with a presentation of the learning objectives followed by a brief introduction to general knowledge in ultrasound physics, basic image interpretation and ultrasound probe handling including orientation, positioning and coupling as well as adequate image amplification and guiding of the patient (“basic skills” in both videos). This was followed by the examination process of the standard views for AU according to the eFAST scheme [42] extended by an organ specific examination of the liver, kidney, spleen and bladder. For TTE, standard views according to the FEEL scheme were shown with emphasis on the parasternal, apical and subcostal views and on particular examination and measurements to be performed (“advanced skills”) [43]. The videos ended with a final summary of all ultrasound standard views. In the two video-tutorials, a brief introduction to general knowledge in ultrasound physics and basic image interpretation was given. The speed could be controlled by the students (double speed, normal speed, jumping backwards). The two-picture design was used with the ultrasound monitor image placed on the left side and the exact corresponding position of the examiner’s hand holding the ultrasound probe located on the right side of the video image. During the production process, the „raw “video material was repeatedly tested before its first use in the study by selected medical students who provided immediate feedback in order to correct for errors and optimize didactics. In addition, both videos were sent to external members from Skills-Labs in Germany, Austria and Switzerland for an a-priori external product-level evaluation. Following their comments, we reduced, simplified and clarified the design and the content of the videos. We further added signaling colors when it seemed to be essential for learning and understanding following the principles of coherence, redundancy, image and signaling. Relevant text, spoken words and visuals were shown synchronically and the description of the presented ultrasound images was clearly addressed according to the dual coding theory, the spatial and temporal contiguity and multimedia principles. We primarily used visuals and spoken words in direct speech by a professional radio speaker with text being only used for clear description of the anatomy captured in the ultrasound images following the modality, personalization and voice principles. **Table S1 in the** Additional file 1 provides a chronological overview on the video and study development process.

**Figures A1 and A2 in the** Additional file 2 show screenshot examples of standard views from both videos. Instructions were provided in a systematic step-by-step manner including anatomical measurements (distances, organ size), physiological measurements (use of motion mode and pulse-wave doppler for valve flow velocity). Importantly, neither pathologies were taught, nor clinical examination findings discussed in the videos.

### Faculty staff-led course without media support

The FS-led course was conducted without any media support under direct supervision of the faculty tutor being a clinical teacher with perennial expertise in both AU and TTE. The faculty tutor consistently taught the identical knowledge as provided in both videos directly using the ultrasound device with one participant as a subject for “live” presentation and within the same time period. Following the segmenting principle, the faculty tutor summarized the learning objectives, continued with each ultrasound standard view followed by a final summary of all ultrasound standard views. In this “live” faculty tutor instruction, further principles of coherence, spatial and temporal contiguity, segmenting, personalization, voice, image and multimedia were consistently applied. During the remaining course period, the faculty tutor otherwise rendered assistance upon request in correspondence to the role of the student tutors in the ViST course. While in the ViST course participants had the opportunity to watch the video (or parts of the video) repeatedly during each two-hour course period, participants of the FS-led course could ask the faculty tutor for repeated information.

### Learning objectives

Both groups were presented with the learning objectives at the beginning of each course, which were introduced for the ViST course in each video and for the FS-led course by the faculty tutor. The learning objectives for both ultrasound courses were identical and divided into 1) general basic skill ultrasound examination including probe handling, image correction and patient guidance and 2) advanced ultrasound examination including standard view adjustment with anatomical structures, physiological measurements and image description. Learning objectives for basic skills were defined as follows:

1a) to apply the correct handling of the convex probe for AU and sector probe for TTE, 1b) to set an adequate image amplification and 1c) to know how to guide the patient on correct positioning for the respective ultrasound examination. Table 1 summarizes the learning objectives for the advanced ultrasound examination skills.

**Table 1:** Learning objectives for advanced abdominal ultrasound and transthoracic echocardiography examination

Standard view	Anatomical structures	Physiological measurements
**Transthoracic echocardiography**		
Parasternal long axis view	Identify left atrium and ventricle, left ventricle outflow tract, aortic valve and mitral valve	Measure thickness of ventricle and septum in motion mode
Parasternal short axis view	Show papillary muscle plane, mitral and aortic valve planes	No measurement required
Apical four-chamber view	Identify all chambers and both atrioventricular valves	Measure flow velocity over mitral valve in pulsed wave doppler mode
**Abdominal ultrasound**		
Liver	Identify liver, portal vein, inferior caval vein, common bile duct	Measure flow velocity in the portal and hepatic vein in pulsed wave doppler mode
Kidney	Identify kidney longitudinal and sagittal	Measure flow velocity in an artery in the parenchyma for calculation of resistance index
Spleen	Identify spleen and hilum	Measure organ size
Bladder	Identify bladder, suprapubic region, uterus/prostate	Measure hilum

### Objective learning outcomes

After completion of all courses, both ultrasound teaching concepts were evaluated by an examination of practical and theoretical skills based on the OSCE (Objective Structured Clinical Examination) format [44]. Each exam was led by one ultrasound-experienced physician blinded to the participants’ course allocation in order to assure group allocation-independent assessment of the students’ performance and limit measurement bias. The OSCEs were conducted on the day after the courses were completed. Training units for both examiners were carried out beforehand to ensure the correct application of the test procedure.

The participants were tested using modified validated examination sheets [45] for each standard view for AU and TTE with predefined fixed evaluation criteria and achievable score points assigned to four main tasks: 1) ultrasound probe handling with the subtasks orientation, positioning, and coupling of the probe as well as adjustment of adequate amplification and patient guidance including special posture or breathing commands (maximum of 10 points); 2) examination skills regarding correct standard view adjustment with anatomical structures (maximum of 12 points) followed by a correct anatomical/physiological measurement typically required for the specific standard view (maximum of six points); 3) image description and explanation, respectively (maximum of four points) and 4) overall performance where the two examiners were asked to assess the overall performance with a maximum of eight points on a numerical evaluation scale according to their subjective impression of the testee. If the testee was unable to accomplish the required subtask on his own within 30 s, either verbal or manual assistance was given by the examiner resulting in a point detraction. For the probe handling and image amplification, the scoring system was uniform for all questionnaires. In order to account for differences in the required patient guidance and degree of difficulty of the measurements and image explanation for each standard view and organ to be performed, the scoring system of these subtasks had to be modified for each questionnaire. As an example, the standard view “bladder” did not require any patient guidance or physiological but only anatomical measurements (i.e. size of bladder). Table 2 shows an example of the questionnaire for the TTE apical four-chamber standard view.

**Table 2:** Exemplary OSCE evaluation sheet for transthoracic echocardiography apical four-chamber view

Task	Task points	Points achieved	Max. Points
1. Probe Handling and patient guidance			**10**
- Orientation			
• Correct or directly checked independently by image movement or coupling	2		
• Corrected after initial difficulties or upon request	1		
• Only with manual assistance	0		
- Positioning			
• Correct, or directly transferred from another view with optimal pivoting	2		
• Corrected after initial difficulties or upon request	1		
• Only with manual assistance	0		
- Coupling			
• Coupling with gel and pressure variation	2		
• Corrected after initial difficulties or upon request	1		
• Missing use of gel or pressure, probe not connected with skin or uncontrolled pressure on xiphoid/ribs	0		
- Adequate amplification			
• Adequate and independent adjustment of amplification	2		
• Corrected after initial difficulties or upon request	1		
• No adequate amplification adjustment despite request	0		
- Guiding of the patient			
• Positioning on the subject’s left side	1		
• Left arm bended beneath head	1		
2. Examination			**18**
- Standard view anatomical structures			
• Correct view (without assistance) of			
o both ventricles, atria and atrioventricular valves	6		
o Perpendicular septum	2		
o Apex in 12 o’clock direction	2		
o Maximum ventricle extension (no foreshortening)	2		
• Correct view only with verbal assistance	4		
• Correct view only with manual assistance	2		
• Standard view cannot be displayed despite manual assistance	0		
- Standard view measurement			
• Correct pulsed wave doppler measurement with			
o sample volume at mitral valve level			
o correct flow profile	2		
• Correct measurement only with manual assistance	2		
• Incorrect measurement despite manual assistance	0		
3. (Correct) Image description/explanation			**4**
- Ventricle left/right	1		
- Interventricular septum	1		
- Aortic and mitral valve	2		
4. Overall Performance			**8**
- Confident	8		
	7		
	6		
	5		
	4		
	3		
	2		
- Marked deficits	1		
**Total score**			**40**

A maximum total score of 40 points could be reached in the OSCE, divided into 32 “objective” points for task parts 1–3, and an additional 8 „subjective” points for overall performance. The additional use of a subjective numerical rating scale ranging from confident to marked deficits was included to increase the validity and reliability as opposed to a single objective scoring system [44, 45]. No pass/fail score was defined for the OSCE. Due to time restrictions for the OSCE, only one out of seven standard views for AU (four standard views) and TTE (three standard views) was tested per student in a simple random fashion.

### Acceptance and subjective learning outcomes

Participants of the ViST course were further asked to evaluate both ultrasound teaching videos using modified questionnaires with closed and open questions based on the evaluation questionnaire of multimedia learning programmes and concepts [46] (**Table A2 in the** Additional file 1**)**. According to the evaluation level model of Kirkpatrick [47], closed questions focused on the general acceptance (reaction level, questions 4, 24, 26 and 27 of the questionnaire) and subjective learning effects (learning level) including motivation (question 2), knowledge expansion (questions 1, 5 and 6) and practical use (questions 8, 21 and 22). Closed questions had to be answered using a rating scale ranging from 1 to 4 with 1: does fully apply, 2: does rather apply, 3: does rather not apply and 4: does not apply. For further analysis, ratings 1 and 2 were summarized and defined as a positive rating. The items technical and content-wise video design were also addressed with closed questions in the questionnaire but not considered for further analysis. Moreover, participants had the opportunity for additional requests to the teaching videos with their response to open questions.

### Statistical analysis

Statistical analysis was performed using GraphPad Prism Version 5.01 for Windows (GraphPad Software, San Diego California USA, www.graphpad.com) and the software R version 3.6.2 [48]. Data are reported as absolute or relative frequencies or as mean and standard deviation.

Differences between groups were tested by a two-factorial ANOVA. The first factor consisted of the two groups (ViST versus FS-led course). The second factor “standard view” had to be added as a covariate being a nominal variable with seven categories, i.e. the 7 ultrasound standard views that were taught in both courses while only one out of seven possible standard views was randomly assigned per student to be examined in the OSCE. This covariate neither showed a significant influence nor significant interaction. Model diagnostic plots showed no signs for deviation from normal distribution or heteroscedasticity. The difference of drop-outs between the two groups was compared with the Fisher exact test. All tests were performed two-sided and a significance level of 0.05 was chosen. Statistical analysis for the secondary objective was performed in a descriptive manner presenting absolute and percentage values of the response to the questionnaire’s scale-rated closed and open questions.

## Results

### Study participants

**Fig. 1:**
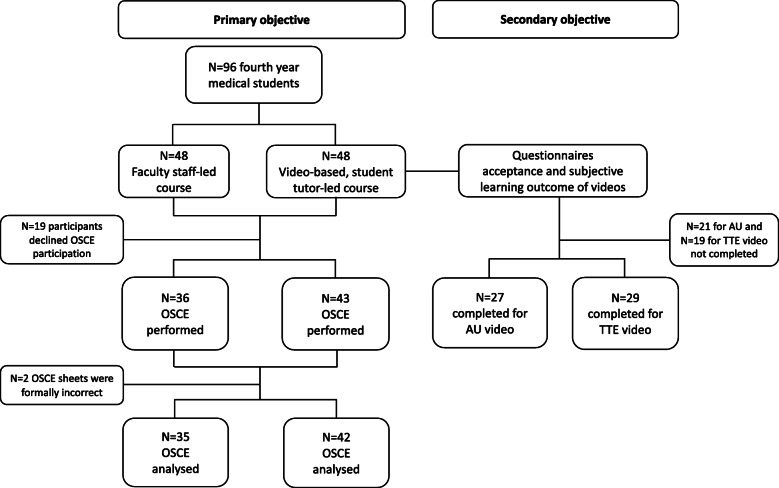
**Study participants flow chart.** Diagram shows the participants flow for the primary objective (objective learning outcomes, Objective Structured Clinical Examination (OSCE)) and secondary objective subjective learning outcomes and learning success of the two ultrasound teaching videos

Figure 1 presents the study participants flow chart. 96 medical students were randomized to either the ViST- or FS-led course (48 students each) with 79 students (75.8%) completing the voluntary OSCE. 77 examination sheets (42 from the ViST- and 35 from the FS course) were formally correct and considered for final statistical analysis. Thus, the number of dropouts for the primary objective was six in the ViST- and 13 in the FS-led course but this difference was not significantly different giving no indication for a potential selection bias (*P* = 0.14). Of the 48 ViST course participants, a total of 27 questionnaires for the AU (64%) and 29 for TTE video (69%), respectively were completed and considered for final evaluation.

### Objective learning outcomes

Table 3 summarizes the results of the OSCE performed for both AU and TTE. There was no significant difference in the mean total point score between the video-based, student tutor and the faculty staff group (video: mean = 31.3, faculty: mean = 32.7, *P* = 0.31). Overall, the standard view “bladder” for AU was examined least while the apical 4-chamber and parasternal long-axis view for TTE were examined most frequently. A stratified analysis for the different distribution of standard views examined in the OSCE showed no significant different means between the two groups.

**Table 3:** Score points achieved in the abdominal ultrasound and transthoracic echocardiography OSCE

Standard view	Total N (%)	Video-based, student tutor-led course		Faculty staff-led course		
		OSCE, N	Total score^a^	OSCE, N	Total score^a^	***P*** value*
Total OSCE	**77 (100)**	**42**	**31.3 ± 6.6**	**35**	**32.7 ± 4.5**	**0.31**
Abdominal ultrasound (AU)						
Total AU OSCE	40 (51.9)	21	31.3 ± 5.5	19	32.8 ± 5.3	n.s.
Portal vein	9 (11.7)	5	29.4 ± 7.9	4	27.5 ± 7.5	n.s.
Kidney	11 (14.3)	5	29.4 ± 4.9	6	33.2 ± 2.3	n.s.
Spleen	12 (15.6)	6	31.5 ± 2.8	6	33.3 ± 2.4	n.s.
Bladder	8 (10.4)	5	35.0 ± 2.1	3	38.0 ± 2.8	n.s.
Transthoracic echocardiography (TTE)						
Total TTE OSCE	37 (48.1)	21	31.3 ± 7.7	16	32.5 ± 3.3	n.s.
Parasternal long axis	11 (14.3)	7	34.7 ± 6.1	4	33.5 ± 1.8	n.s.
Parasternal short axis	13 (16.9)	7	32.8 ± 7.8	6	38.0 ± 3.4	n.s.
Apical 4-chamber view	13 (16.9)	7	29.0 ± 7.3	6	30.5 ± 2.9	n.s.

* ANOVA analysis for comparison of total point score between the video-based, student tutor and faculty staff course

^a^ Total score achieved as mean ± SD

*AU* abdominal ultrasound; *n.s.* not significant; *TTE* transthoracic echocardiography

**Table A3 in the** Additional file 1 shows the comparison of the scores achieved in each of the basic and advanced skill subtasks of the OSCE between the two courses. As different scores for the respective subtasks could be obtained depending on the standard view and difficulty of measurements, the results are given as percentage of each maximum sub-task and total score to be achieved, respectively.

### Acceptance and subjective learning outcomes

**Fig. 2:**
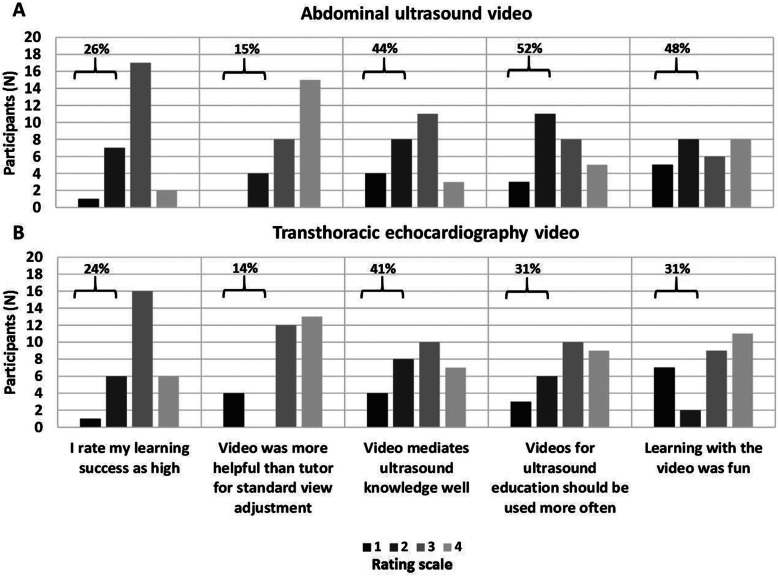
**Acceptance (reaction level) and motivation of the ultrasound teaching videos.** The questionnaire’s results for acceptance (reaction level, questions 2, 4, 7, 26 and 27 of the questionnaire) and motivation (learning level, question 2) are shown according to the response rating scale 1 (does fully apply) to 4 (does not apply) for the abdominal ultrasound video (Panel A) and transthoracic echocardiography video (Panel B). The positive ratings (summarized ratings 1 and 2) are outlined in the diagram as percentage of the completed questionnaires (29 for the transthoracic echocardiography and 27 for the abdominal ultrasound video)

**Fig. 3:**
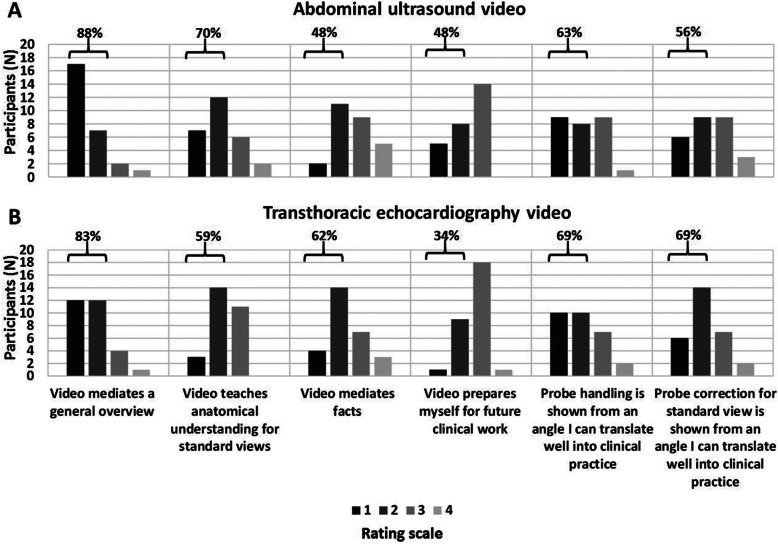
**Subjective learning success of the ultrasound teaching videos.** The questionnaire’s results for subjective learning success (knowledge expansion, questions 1, 5 and 6 of the questionnaire and practical use, questions 8, 21 and 22 of the questionnaire) are shown according to the response rating scale 1 (does fully apply) to 4 (does not apply) for the abdominal ultrasound video (Panel A) and transthoracic echocardiography video (Panel B). The positive ratings (summarized ratings 1 and 2) are outlined in the diagram as percentage of the completed questionnaires (29 for the transthoracic echocardiography and 27 for the abdominal ultrasound video)

Figure 2 summarizes the results for acceptance and learning motivation with positive ratings of 15–48% for the AU and 14–41% for the TTE video. For knowledge expansion and practical use of both teaching videos, positive ratings ranged from 48 to 88% and 34–83% of the answered questions, respectively (Fig. 3).

**Fig. 4:**
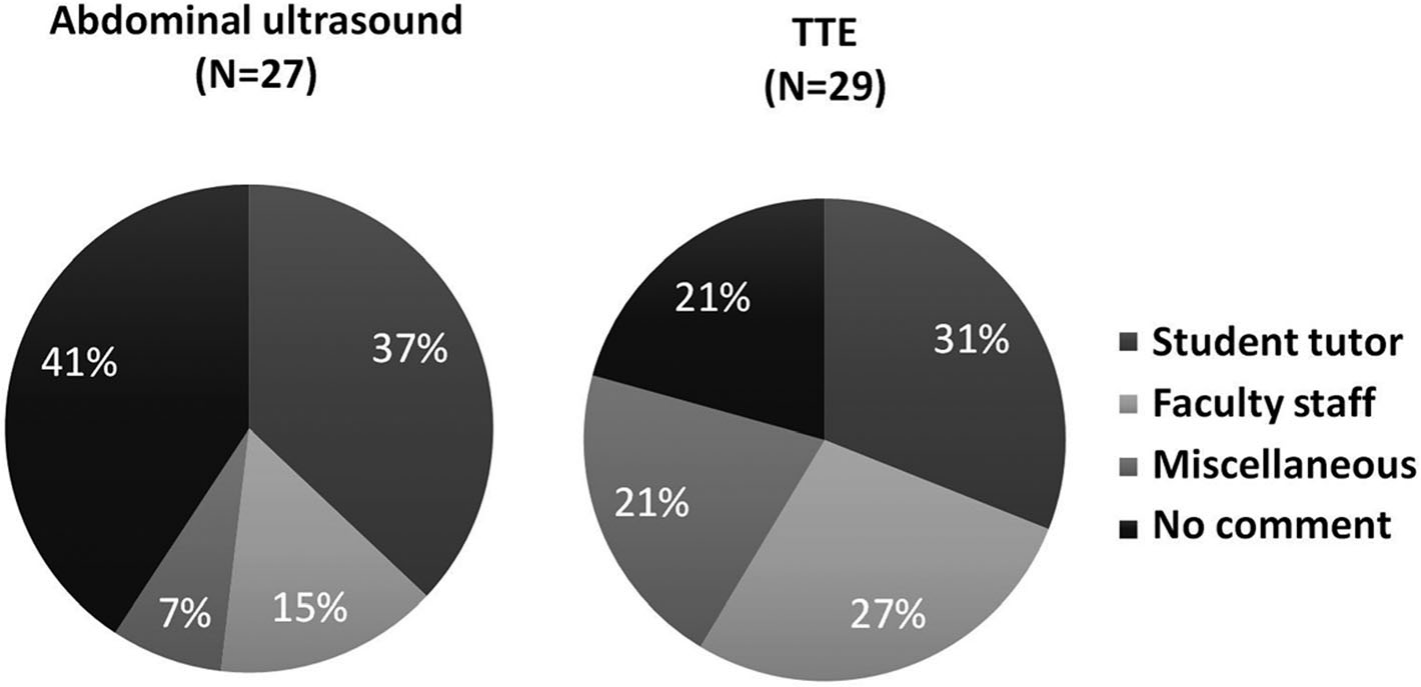
**Additional requests to the teaching videos.** Diagrams show the relative distribution (as percent) of additional requests to either the abdominal ultrasound (left) or transthoracic echocardiography (TTE, right) teaching video based on the response to open questions in the questionnaire. 27 questionnaires were filled out for abdominal ultrasound, 29 for TTE. Miscellaneous comments included more time for watching the videos (two participants in the abdominal ultrasound and one participant in the TTE video course), a power point presentation instead of a video (one participant in the TTE video), and the use of an anatomic heart model in addition to the TTE video (three participants in the TTE course)

According to open comments in each questionnaire, 14 participants for the AU and 17 participants for the TTE video mentioned that either the student or faculty tutor would be necessary in addition to the video as immediate assistance was viewed as necessary and helpful for completing the practical skills. Miscellaneous comments included more time for watching the videos (three participants), a power point presentation instead of a video (one participant), and the use of an anatomic heart model in addition to the video (three participants) (Fig. 4).

## Discussion

In this prospective randomized, single-blinded study, a ViST course for AU and TTE was compared with a FS-led course without any media support in terms of objective learning outcomes for undergraduate medical students without previous knowledge in ultrasound. The mean total point score achieved in 77 completed OSCE was non-significantly different with 31.3 points in the ViST and 32.7 in the FS-led course. No significant differences were found in any of the seven standard views examined in the OSCE. While acceptance of both videos and motivation was rated generally low, subjective learning success, however, was rated positively in up to 88% of the responses. Supervision of the student or faculty tutor, respectively was still deemed necessary in addition to the teaching videos by more than half of the responding participants of the ViST course.

Modern approaches to medical teaching and learning have emerged in recent years drifting away from teacher-centered (passive) to learner-centered (active) styles in order to increase student engagement and essentially increase learning outcomes [15, 49, 50]. Ultrasound curricula naturally encompass active learning principles including self-directed, collaborative learning when practical skills are taught hands-on in a student team alternating in the role of examiner and patient under supervision of faculty staff [3, 8]. As such, our department has established an extracurricular FS-led ultrasound course that was lately constantly overbooked by popular demand and shortness of expert clinical ultrasound teachers. To overcome this barrier and increase course availability, we combined a video-based ultrasound instruction and peer-teaching in a new course concept which has not been evaluated thus far. Peer-teaching concepts alone have already been shown to be feasible and effective for teaching basic as well as advanced POCUS skills compared to faculty staff [17–20].

The use of digital media, video instruction and online formats (e-learning) as a contemporary way of teaching appears to be particularly suitable [24, 37, 38], not only due to the ongoing COVID-19 pandemic where university faculties have to adapt to these alternative educational strategies [29, 51]. A Korean nationwide survey revealed that teaching videos increased students’ subjective feelings of competence and were rated to be useful in different medical disciplines [25]. With respect to objective learning outcomes, studies comparing video-based against traditional teaching methods have reported divergent results with either improved theoretical practical knowledge [37, 52, 53], no effect differences [35] or even worse learning outcomes [36]. With respect to video-based ultrasound teaching, only two studies yet exist that have evaluated objective learning outcomes or students’ acceptance, respectively. Altersberger et al. [41] aimed at evaluating students’ perceptions of different ultrasound teaching videos illustrating a predefined examination process, image optimization, and nine standardized ultrasound views as compared to other learning materials (live demonstration by an instructor, hands-on training, written materials). In the videos, tutors demonstrated the steps needed to perform standard ultrasound views and to identify anatomical landmarks. The videos were made available to fourth year medical students in preparation for an ultrasound OSCE and 119 of 134 students responding to their questionnaire rated the instructional videos as very helpful [41]. Overall, the videos were perceived as the second most helpful learning material after “self-execution and feedback” which is in contrast to the rather low acceptance of the two ultrasound teaching videos in our study. Gradl-Dietsch and coworkers [40] compared the effect of different teaching approaches for TTE on learning outcomes including peer teaching, peer teaching using Peyton’s four-step approach, team-based and video-based learning. In the video-based only learning group, ultrasound device handling skills and standard view acquisition were presented followed by practical hands-on training without supervision. Although the majority of the participating 79 students achieved good objective results on theoretical and practical skills, the acceptance particularly of the sole video-based teaching was low as compared to the other three concepts. While there was no difference in the overall rating of the course or assessment results between types of intervention, students in the video group would have generally preferred a different teaching method with a preference for medical expert- instead of peer tutor education. This is fully in line with our study results although we observed no preference for either the faculty- or student tutor but were further able to show that subjective gain in knowledge was rated positively by the majority of participants. Gradl-Dietsch et al. attributed their results to the fact that the video was not permanently made available to the students and that the demonstrated skills were presumably much more complex and challenging. Thus, they proposed that the possibility of video review at home may have improved acceptance. Only three participants in our study mentioned that more time for the ultrasound videos was required beyond the presentation time in each two-hour course. As opposed to the two other studies not explicitly referencing any multimedia design principle, the videos used in our study followed 11 of the 12 learning principles according to Mayer [30, 31]. Before first use in our study, the videos were repeatedly tested by selected medical students in order to optimize didactics specifically required by the target audience of ultrasound unexperienced medical students. Implementation of these principles aim at reducing cognitive load and enhancing learning which has been shown to improve objective learning outcomes when compared to traditional lectures [33, 34, 52]. However, one may speculate that the design principles may rather influence objective than subjective learning outcomes and acceptance among individuals with different learning preferences, respectively [52, 54]. Other digital formats such as mobile learning applications (“app”) or adaptive computer supported collaborative e-learning systems may be associated with a higher general acceptance [55, 56]. Lai and coworkers recently showed that the incorporation of game design elements (“gamification approach”) in POCUS training was not only effective in terms of practical skill acquisition but was also associated with an increased engagement and enjoyment among junior doctors [57].

Our study has limitations to be addressed. The main limitation is that we did not control for extracurricular sources of studying and training right before the OSCE. We are therefore unable to exclude potential bias that participants of either group had a knowledge advantage during the study period potentially washing out any effects of the intervention. Albeit only students without preexisting knowledge were enrolled in our study and the randomization performed tends to limit this bias across groups, ideally for the study purpose there should have been an unannounced assessment of knowledge to know if students remember without the potential influence of restudy. Second, the OSCEs were performed shortly after both courses were completed, so information on long-term retention of both theoretical and practical ultrasound skills cannot be deduced from our study. Third, we only included a relatively small cohort of fourth-year medical students including drop-outs for both the primary and secondary objective. The difference in drop-outs was not statistically significant between the ViST- and FS-led course. Thus, no indication for a significant selection bias for the primary objective was found. We are unable to finally rule out a possible response bias for the secondary objective between the AU and TTE video group as the anonymous data collection inhibited the performance of the required McNemar’s statistical test which necessitates matched pairs, hence limiting the internal validity of our study. Moreover, acceptance and subjective learning outcomes were only evaluated for ViST but not in comparison to the FS-led course as we did for the primary objective. Hence, we are unable to report results for the comparison of the student versus faculty tutor performance and acceptance of the FS-led course within our study setting. The “classical” FS-led course as the established “standard practice” has been shown to constantly receive positive student ratings in the regular term-wise teaching evaluation. Thus, in the planning of our study we inferred that this classical FS-led course had a high acceptability and deemed to only evaluate acceptance and subjective learning outcomes of the “new” ViST course format. With respect to tutor performance, aforementioned studies have already shown that trained students gain acceptance as ultrasound peer tutors [18, 19]. Comments made in our questionnaires from the ViST course participants revealed that either the student or faculty tutor - without preference for one or the other - was still deemed necessary in addition to the video instruction. Due to time restrictions of the OSCE and availability of examiners, we were only able to test one out of seven standard views for AU and TTE per student by one blinded examiner for each OSCE which may have influenced the reliability and validity of the OSCE [58]. In order to keep this potential influence on reliability small, we followed the necessary interventions during the OSCE with precise timing instructions as described by Hofer et al. [45]. Our statistical analyses showed no influence of the chosen standard view on the outcome and no interaction with group status. In addition, training units for both blinded examiners were carried out to ensure the correct application of the test procedure. Lastly, due to the anonymous data analysis we did not document demographic characteristics of the participants including detailed information on age (other than fourth year medical students) and gender allocation to both courses. Therefore, we are unable to report potential effects of the participants’ characteristics on both study objectives. However, Gradl-Dietsch et al. did not find significantly different effects of gender on basic echocardiographic skills comparing four different peer-teaching concepts [40].

The strength of our study is that it is – to our knowledge – the first evaluation to date on the effect of a combined video-based, student tutor teaching approach on objective as well as subjective learning outcomes and acceptance in a real-life university educational setting.

## Conclusions

Our study shows that the combination of video-based, peer-teaching by trained student tutors resulted in similar objective and subjective knowledge acquisition in basic and advanced AU and TTE as opposed to a faculty staff-led concept without media support. Acceptance and learning motivation of both teaching videos alone was rather limited and support from either student or faculty tutors was still deemed necessary by the participants. Nevertheless, the combined video-based, student tutor concept offers the possibility to meet the increased demand for undergraduate ultrasound education and allow for greater flexibility for both faculty staff and university infrastructure resource allocation. Future studies are warranted to test this concept in a larger number of students including the teaching of pathologies**.**

## Supplementary Information

**Additional file 1 Table A1**. Overview on the development and evaluation process of the video-based, student tutor-led course. **Table A2**. Questionnaire for subjective learning success and acceptance of the teaching video exemplary for the transthoracic echocardiography video. Participants’ subjective learning success and acceptance of the teaching videos was evaluated using modified questionnaires with scale-rated closed (from 1 does fully apply to 4 does not apply) and open questions based on Kirkpatrick’s evaluation level model. **Table A3**. Comparison of the score points achieved in the OSCE tasks between the video-based, student tutor and the faculty staff-led course.

**Additional file 2 Figure A1**. Screen shot example from the abdominal ultrasound video. Panel A shows ultrasound probe position for the standard view of the liver. Panel B shows schematic probe position for this view as presented in the video. Panel C shows obtained ultrasound image from this view. Panel D shows the same ultrasound image including labeling of anatomic landmarks to be identified in this standard view. **Figure A2**. Screen shot example from the TTE video. Panel A shows screen shot for overview thorax anatomy and 3 respective transthoracic echocardiography (TTE) standard views. Panel B shows probe handling and patient positioning for standard view parasternal long-axis. Panel C shows the obtained ultrasound image of the heart from this standard view. Panel D shows the corresponding schematic drawing with the right (RV) and left ventricle (LV), interventricular septum (IVS), aortic valve (AK) und left atrium (LA) as presented in the video.

## Data Availability

The datasets used and/or analyzed during the present study are available from the corresponding author on reasonable request.

## Abbreviations

AU: Abdominal ultrasound
eFAST: Extended Focused Assessment with Sonography for Trauma
FEEL: Focused Echocardiography in Emergency Life support
FS: Faculty staff
n.s.: Not significant
OSCE: Objective Structured Clinical Examination
POCUS: Point-of-care ultrasound
TTE: Transthoracic echocardiography
ViST: Video-based, student tutor
WFUMB: World Federation of Ultrasound in Medicine and Biology

## References

[1] Tarique U, Tang B, Singh M, Kulasegaram KM, Ailon J (2018). Ultrasound curricula in undergraduate medical education: a scoping review. J Ultrasound Med.

[2] Wolf R, Geuthel N, Gnatzy F, Rotzoll D (2019). Undergraduate ultrasound education at German-speaking medical faculties: a survey. GMS J Med Educ.

[3] Hoppmann RA, Rao VV, Bell F, Poston MB, Howe DB, Riffle S, Harris S, Riley R, McMahon C, Wilson LB, Blanck E, Richeson NA, Thomas LK, Hartman C, Neuffer FH, Keisler BD, Sims KM, Garber MD, Shuler CO, Blaivas M, Chillag SA, Wagner M, Barron K, Davis D, Wells JR, Kenney DJ, Hall JW, Bornemann PH, Schrift D, Hunt PS, Owens WB, Smith RS, Jackson AG, Hagon K, Wilson SP, Fowler SD, Catroppo JF, Rizvi AA, Powell CK, Cook T, Brown E, Navarro FA, Thornhill J, Burgis J, Jennings WR, JB MC, Nottingham JM, Kreiner J, Haddad R, Augustine JR, Pedigo NW, Catalana PV (2015). The evolution of an integrated ultrasound curriculum (iUSC) for medical students: 9-year experience. Crit Ultrasound J.

[4] Hoffmann B, Blaivas M, Abramowicz J, Bachmann M, Badea R, Braden B, Cantisani V, Chammas MC, Cui XW, Dong Y, Gilja OH, Hari R, Lamprecht H, Nisenbaum H, Nolsoe CP, Nurnberg D, Prosch H, Radzina M, Recker F, Sachs A, Saftoiu A, Serra A, Vinayak S, Westerway S, Chou YH, Dietrich CF (2020). Medical student ultrasound education, a WFUMB position paper, part II. A consensus statement of ultrasound societies. Med Ultrason.

[5] Dietrich CF, Hoffmann B, Abramowicz J, Badea R, Braden B, Cantisani V, Chammas MC, Cui XW, Dong Y, Gilja OH, Hari R, Nisenbaum H, Nicholls D, Nolsoe CP, Nurnberg D, Prosch H, Radzina M, Recker F, Sachs A, Saftoiu A, Serra A, Sweet L, Vinayak S, Westerway S, Chou YH, Blaivas M (2019). Medical student ultrasound education: a WFUMB position paper. Part I Ultrasound Med Biol.

[6] Hofer M, Kamper L, Miese F, Kropil P, Naujoks C, Handschel J, Heussen N (2012). Quality indicators for the development and didactics of ultrasound courses in continuing medical education. Ultraschall Med.

[7] Heinzow HS, Friederichs H, Lenz P, Schmedt A, Becker JC, Hengst K, Marschall B, Domagk D (2013). Teaching ultrasound in a curricular course according to certified EFSUMB standards during undergraduate medical education: a prospective study. BMC Med Educ.

[8] Patel SG, Benninger B, Mirjalili SA (2017). Integrating ultrasound into modern medical curricula. Clin Anat.

[9] Parmelee D, Michaelsen LK, Cook S, Hudes PD (2012). Team-based learning: a practical guide: AMEE guide no. 65. Med Teach.

[10] Rosen MA, DiazGranados D, Dietz AS, Benishek LE, Thompson D, Pronovost PJ, Weaver SJ (2018). Teamwork in healthcare: key discoveries enabling safer, high-quality care. Am Psychol.

[11] Cawthorn TR, Nickel C, O'Reilly M, Kafka H, Tam JW, Jackson LC, Sanfilippo AJ, Johri AM (2014). Development and evaluation of methodologies for teaching focused cardiac ultrasound skills to medical students. J Am Soc Echocardiogr.

[12] Turner EE, Fox JC, Rosen M, Allen A, Rosen S, Anderson C (2015). Implementation and assessment of a curriculum for bedside ultrasound training. J Ultrasound Med.

[13] Yu TC, Wilson NC, Singh PP, Lemanu DP, Hawken SJ, Hill AG (2011). Medical students-as-teachers: a systematic review of peer-assisted teaching during medical school. Adv Med Educ Pract.

[14] Ten Cate O, Durning S (2007). Dimensions and psychology of peer teaching in medical education. Med Teach.

[15] Ross MT, Cameron HS (2007). Peer assisted learning: a planning and implementation framework: AMEE guide no. 30. Med Teach.

[16] Burgess A, McGregor D (2018). Peer teacher training for health professional students: a systematic review of formal programs. BMC Med Educ.

[17] Knobe M, Munker R, Sellei RM, Holschen M, Mooij SC, Schmidt-Rohlfing B, Niethard FU, Pape HC (2010). Peer teaching: a randomised controlled trial using student-teachers to teach musculoskeletal ultrasound. Med Educ.

[18] Celebi N, Zwirner K, Lischner U, Bauder M, Ditthard K, Schurger S, Riessen R, Engel C, Balletshofer B, Weyrich P (2012). Student tutors are able to teach basic sonographic anatomy effectively - a prospective randomized controlled trial. Ultraschall Med.

[19] Celebi N, Griewatz J, Malek NP, Krieg S, Kuehnl T, Muller R, Pauluschke-Frohlich J, Debove I, Riessen R, Zipfel S, Frohlich E (2019). Development and implementation of a comprehensive ultrasound curriculum for undergraduate medical students - a feasibility study. BMC Med Educ.

[20] Ahn JS, French AJ, Thiessen ME, Kendall JL (2014). Training peer instructors for a combined ultrasound/physical exam curriculum. Teach Learn Med.

[21] Kuhl M, Wagner R, Bauder M, Fenik Y, Riessen R, Lammerding-Koppel M, Gawaz M, Fateh-Moghadam S, Weyrich P, Celebi N (2012). Student tutors for hands-on training in focused emergency echocardiography--a randomized controlled trial. BMC Med Educ.

[22] Sono4students: https://sono4students.uni-bonn.de. Last accessed on Aug 8, 2020.

[23] Tang B, Coret A, Qureshi A, Barron H, Ayala AP, Law M (2018). Online lectures in undergraduate medical education: scoping review. JMIR Med Educ.

[24] Prober CG, Khan S (2013). Medical education reimagined: a call to action. Acad Med.

[25] Jang HW, Kim KJ (2014). Use of online clinical videos for clinical skills training for medical students: benefits and challenges. BMC Med Educ.

[26] Hempel D, Haunhorst S, Sinnathurai S, Seibel A, Recker F, Heringer F, Michels G, Breitkreutz R (2016). Social media to supplement point-of-care ultrasound courses: the "sandwich e-learning" approach. A randomized trial. Crit Ultrasound J.

[27] Ellaway R, Masters K (2008). AMEE guide 32: e-learning in medical education part 1: learning, teaching and assessment. Med Teach.

[28] 123sonography: https://www.123sonography.com. Last accessed on Aug 8, 2020.

[29] Rose S (2020). Medical student education in the time of COVID-19. JAMA..

[30] Mayer RE (2010). Applying the science of learning to medical education. Med Educ.

[31] Mayer RE (2008). Applying the science of learning: evidence-based principles for the design of multimedia instruction. Am Psychol.

[32] Mayer RE (2020). Designing multimedia instruction in anatomy: an evidence-based approach. Clin Anat.

[33] Issa N, Schuller M, Santacaterina S, Shapiro M, Wang E, Mayer RE, DaRosa DA (2011). Applying multimedia design principles enhances learning in medical education. Med Educ.

[34] Issa N, Mayer RE, Schuller M, Wang E, Shapiro MB, DaRosa DA (2013). Teaching for understanding in medical classrooms using multimedia design principles. Med Educ.

[35] Weber U, Constantinescu MA, Woermann U, Schmitz F, Schnabel K (2016). Video-based instructions for surgical hand disinfection as a replacement for conventional tuition? A randomised, blind comparative study. GMS J Med Educ.

[36] Platz E, Liteplo A, Hurwitz S, Hwang J (2011). Are live instructors replaceable? Computer vs. classroom lectures for EFAST training. J Emerg Med.

[37] Maloney S, Storr M, Paynter S, Morgan P, Ilic D (2013). Investigating the efficacy of practical skill teaching: a pilot-study comparing three educational methods. Adv Health Sci Educ Theory Pract.

[38] Lumlertgul N, Kijpaisalratana N, Pityaratstian N, Wangsaturaka D (2009). Cinemeducation: a pilot student project using movies to help students learn medical professionalism. Med Teach.

[39] Alqahtani ND, Al-Jewair T, Al-Moammar K, Albarakati SF, EA AL. (2015). Live demonstration versus procedural video: a comparison of two methods for teaching an orthodontic laboratory procedure. BMC Med Educ.

[40] Gradl-Dietsch G, Menon AK, Gursel A, Gotzenich A, Hatam N, Aljalloud A, Schrading S, Holzl F, Knobe M (2018). Basic echocardiography for undergraduate students: a comparison of different peer-teaching approaches. Eur J Trauma Emerg Surg.

[41] Altersberger M, Pavelka P, Sachs A, Weber M, Wagner-Menghin M, Prosch H (2019). Student perceptions of instructional ultrasound videos as preparation for a practical assessment. Ultrasound Int Open.

[42] Pace J, Arntfield R (2018). Focused assessment with sonography in trauma: a review of concepts and considerations for anesthesiology. Can J Anaesth.

[43] Breitkreutz R, Walcher F, Seeger FH (2007). Focused echocardiographic evaluation in resuscitation management: concept of an advanced life support-conformed algorithm. Crit Care Med.

[44] Walzak A, Bacchus M, Schaefer JP, Zarnke K, Glow J, Brass C, McLaughlin K, Ma IW (2015). Diagnosing technical competence in six bedside procedures: comparing checklists and a global rating scale in the assessment of resident performance. Acad Med.

[45] Hofer M, Kamper L, Sadlo M, Sievers K, Heussen N (2011). Evaluation of an OSCE assessment tool for abdominal ultrasound courses. Ultraschall Med.

[46] Stöckl M, Straka GA, Schenkel P, Holz H (1995). Evaluation eines Lernprogramms für die CAD-Weiterbildung älterer Arbeitnehmer und Arbeitnehmerinnen - Design und erste Ergebnisse. Evaluation multimedialer Lernprogramme und Lernkonzepte: Berichte aus der Berufsbildungspraxis.

[47] Kirkpatrick DL, Kirkpatrick JD (2006). Evaluating training programs. The four levels.

[48] R: A language and environment for statistical computing. R Foundation for Statistical Computing: https://www.R-project.org/. Last accessed on Aug 8, 2020.

[49] Taylor DC, Hamdy H (2013). Adult learning theories: implications for learning and teaching in medical education: AMEE guide no. 83. Med Teach.

[50] McCoy L, Pettit RK, Kellar C, Morgan C (2018). Tracking active learning in the medical school curriculum: a learning-centered approach. J Med Educ Curric Dev.

[51] Schlenz MA, Schmidt A, Wostmann B, Kramer N, Schulz-Weidner N (2020). Students' and lecturers' perspective on the implementation of online learning in dental education due to SARS-CoV-2 (COVID-19): a cross-sectional study. BMC Med Educ.

[52] Yiu SHM, Spacek AM, Pageau PG, Woo MYC, Curtis Lee A, Frank JR (2020). Dissecting the contemporary clerkship: theory-based educational trial of videos versus lectures in medical student education. AEM Educ Train.

[53] Schwerdtfeger K, Wand S, Schmid O, Roessler M, Quintel M, Leissner KB, Russo SG (2014). A prospective, blinded evaluation of a video-assisted '4-stage approach' during undergraduate student practical skills training. BMC Med Educ.

[54] Woodham LA, Ellaway RH, Round J, Vaughan S, Poulton T, Zary N (2015). Medical student and tutor perceptions of video versus text in an interactive online virtual patient for problem-based learning: a pilot study. J Med Internet Res.

[55] Taveira-Gomes T, Saffarzadeh A, Severo M, Guimaraes MJ, Ferreira MA (2014). A novel collaborative e-learning platform for medical students - ALERT STUDENT. BMC Med Educ.

[56] Fernandez-Lao C, Cantarero-Villanueva I, Galiano-Castillo N, Caro-Moran E, Diaz-Rodriguez L, Arroyo-Morales M (2016). The effectiveness of a mobile application for the development of palpation and ultrasound imaging skills to supplement the traditional learning of physiotherapy students. BMC Med Educ.

[57] Lai AKH, Noor Azhar AMB, Bustam AB, Tiong XT, Chan HC, Ahmad RB, Chew KS (2020). A comparison between the effectiveness of a gamified approach with the conventional approach in point-of-care ultrasonographic training. BMC Med Educ.

[58] Harden RM (2016). Revisiting 'Assessment of clinical competence using an objective structured clinical examination (OSCE)'. Med Educ.

