# Supplementary material for: Video-based, student tutor- versus faculty staff-led ultrasound course for medical students – a prospective randomized study
Source: BMC Med Educ. 2020 Dec 16;20:512. doi: 10.1186/s12909-020-02431-8 (PMC7741871; doi:10.1186/s12909-020-02431-8)
Supplement: Supplementary file 1 — Additional file 1 Table A1. Overview on the development and evaluation process of the video-based, student tutor-led course. Table A2. Questionnaire for subjective learning success and acceptance of the teaching video exemplary for the transthoracic echocardiography video. Participants’ subjective learning success and acceptance of the teaching videos was evaluated using modified questionnaires with scale-rated closed (from 1 does fully apply to 4 does not apply) and open questions based on Kirkpatrick’s evaluation level model. Table A3. Comparison of the score points achieved in the OSCE tasks between the video-based, student tutor and the faculty staff-led course. [file 12909_2020_2431_MOESM1_ESM.pdf]

Additional File 1

To: Video-based, student tutor- versus faculty staff-led ultrasound tutorial for medical students – a prospective randomized study

BMC Medical Education

Christine Eimer, MD[1], Max Duschek, MD[1], Andreas Emanuel Jung[1], MD[1], Günther Zick MD[1], Amke Caliebe, PhD[2], Matthias Lindner, MD[1], Norbert Weiler, MD[1] and Gunnar Elke, MD[1]

1 Department of Anaesthesiology and Intensive Care Medicine, University Medical Center Schleswig-Holstein, Campus Kiel, Kiel, Germany

2 Institute of Medical Informatics and Statistics, Christian-Albrechts-University Kiel, University Medical Center Schleswig-Holstein, Campus Kiel, 24105 Kiel, Germany

Index Additional File 1

Table A1. Overview on the development and evaluation process of the video-based, student tutor-led course........2

Table A2. Questionnaire for subjective learning success and acceptance of the teaching video exemplary for the transthoracic echocardiography video........3

Table A3. Comparison of the score points achieved in the OSCE tasks between the video-based, student tutor and faculty staff-led course. 5

Table A1. Overview on the development and evaluation process of the video-based, student tutor-led course

Development phase	Film production phase	Course and evaluation phase
• Teaching two student tutors ultrasound techniques<br>○ Theoretical and practical training by two experienced sonographers for ultrasound skills as required for the course<br>• Establish concept for the film<br>• Prepare film script	• Shooting<br>• Postproduction<br>• Evaluation by medical students and colleagues from other European Skills Labs	• Testing of the small group courses with collegiate tutors and supervision<br>• OSCE audit<br>• Film evaluation
**Period 8 months**	**Period 8 months**	**Period 4 months**

Table A2. Questionnaire for subjective learning success and acceptance of the teaching video exemplary for the transthoracic echocardiography video

**Questionnaire Video – Transthoracic echocardiography**				
	**Does fully apply**	**Does rather apply**	**Does rather not apply**	**Does not apply**
**Learning with the video**	**1**	**2**	**3**	**4**
1. The video mediates a general overview on abdominal ultrasound	☐	☐	☐	☐
2. Learning with the video was fun	☐	☐	☐	☐
3. The video shows practical skills	☐	☐	☐	☐
4. I rate my learning success as high	☐	☐	☐	☐
5. The video teaches an anatomical understanding for the standard views	☐	☐	☐	☐
6. The video mediates facts	☐	☐	☐	☐
7. I have learned more from the tutor than from the video	☐	☐	☐	☐
8. The video prepares myself for my future clinical work	☐	☐	☐	☐
9. The video content is too difficult	☐	☐	☐	☐
10. Much information is superfluous	☐	☐	☐	☐
11. The video has many unexplained or unknown terms	☐	☐	☐	☐
12. The speaker's text corresponds with the video image	☐	☐	☐	☐
13. The pace of the speaker is pleasant	☐	☐	☐	☐
14. Essential content for understanding is shown	☐	☐	☐	☐
15. Essential content for practical standard view adjustment is shown	☐	☐	☐	☐
16. The order of content is reasonable	☐	☐	☐	☐
17. Graphics/figures in the video were helpful	☐	☐	☐	☐
18. The video has too little graphics/figures	☐	☐	☐	☐
19. Video sequences, ultrasound images and graphics are clear	☐	☐	☐	☐
20. Legends in the video were readable	☐	☐	☐	☐

21. Probe handling is shown from an angle so that I can translate it well into clinical practice	☐	☐	☐	☐
22. Probe correction for standard view is shown from an angle so that I can translate it well into clinical practice	☐	☐	☐	☐
23. Probe movements are well represented by the camera settings of the video	☐	☐	☐	☐
24. The video was more helpful for standard view adjustment than the tutor	☐	☐	☐	☐
25. Control of software for playing the video is convenient	☐	☐	☐	☐
26. Ultrasound skills are mediated well by the video	☐	☐	☐	☐
27. Videos should be used more often for ultrasound teaching	☐	☐	☐	☐
**Representation of standard views in the video**				
28. The video is detailed enough	☐	☐	☐	☐
29. Description and representation are understandable	☐	☐	☐	☐
30. I have understood the use of M-mode	☐	☐	☐	☐
31. I have understood the use of PW-doppler and measurement of mitral valve flow velocity	☐	☐	☐	☐
32. I have understood the use of PW-doppler and measurement of aortic valve flow velocity	☐	☐	☐	☐
**Open questions**				
33. What did I like most in the video?				
34. Can the video be improved?				
35. Apart from the video I would like to have the following aids:				

Table A3. Comparison of the score points achieved in the OSCE tasks between the video-based, student tutor and faculty staff-led course.

OSCE audit	Video-based, student tutor-led course (%)	Faculty staff-led course (%)
**Maximum score points, N**	**40**	**40**
**Total score points, %**	**78.3**	**81.6**
**Basic skills, %**		
Orientation of the probe	80.9	82.9
Positioning of the probe	75.0	75.7
Coupling of the probe	97.6	95.7
Adequate amplification	80.9	84.3
Guiding of the patient	73.9	77.1
**Advanced skills, %**		
Standard view representation	79.0	82.7
Measurement	76.2	81.0
Image description/ explanation	82.1	91.4
**Overall Performance, %**	75.9	78.6

Score points achieved in each task are shown as percentage of the maximum score (40 points).
