# Supplementary material for: Video-based, student tutor- versus faculty staff-led ultrasound course for medical students – a prospective randomized study
Source: BMC Med Educ. 2020 Dec 16;20:512. doi: 10.1186/s12909-020-02431-8 (PMC7741871; doi:10.1186/s12909-020-02431-8)
Supplement: Supplementary file 2 — Additional file 2 Figure A1. Screen shot example from the abdominal ultrasound video. Panel A shows ultrasound probe position for the standard view of the liver. Panel B shows schematic probe position for this view as presented in the video. Panel C shows obtained ultrasound image from this view. Panel D shows the same ultrasound image including labeling of anatomic landmarks to be identified in this standard view. Figure A2. Screen shot example from the TTE video. Panel A shows screen shot for overview thorax anatomy and 3 respective transthoracic echocardiography (TTE) standard views. Panel B shows probe handling and patient positioning for standard view parasternal long-axis. Panel C shows the obtained ultrasound image of the heart from this standard view. Panel D shows the corresponding schematic drawing with the right (RV) and left ventricle (LV), interventricular septum (IVS), aortic valve (AK) und left atrium (LA) as presented in the video. [file 12909_2020_2431_MOESM2_ESM.pdf]

Additional File 2

To: Video-based, student tutor- versus faculty staff-led ultrasound tutorial for medical students – a prospective randomized study

BMC Medical Education

Christine Eimer, MD[1], Max Duschek, MD[1], Andreas Emanuel Jung, MD[1], Günther Zick MD[1], Amke Caliebe, PhD[2], Matthias Lindner, MD[1], Norbert Weiler, MD[1] and Gunnar Elke, MD[1]

1 Department of Anaesthesiology and Intensive Care Medicine, University Medical Center Schleswig-Holstein, Campus Kiel, Kiel, Germany

2 Institute of Medical Informatics and Statistics, Christian-Albrechts-University Kiel, University Medical Center Schleswig-Holstein, Campus Kiel, 24105 Kiel, Germany

Index Additional File 2

Figure A1. Screen shot example from the abdominal ultrasound video..2

Figure A2. Screen shot example from the TTE video 3

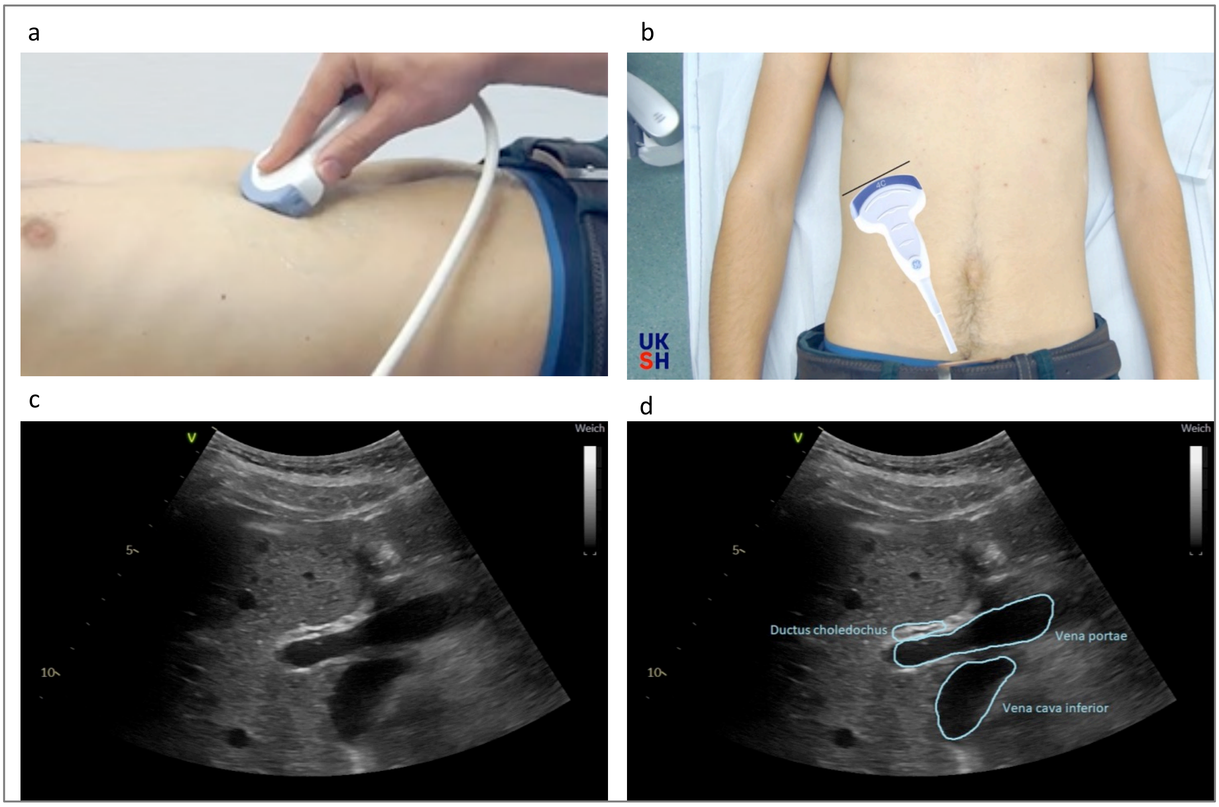

Figure A1. Screen shot example from the abdominal ultrasound video

Panel a shows ultrasound probe position for the standard view of the liver. Panel b shows schematic probe position for this view as presented in the video. Panel c shows obtained ultrasound image from this view. Panel d shows the same ultrasound image including labeling of anatomic landmarks to be identified in this standard view.

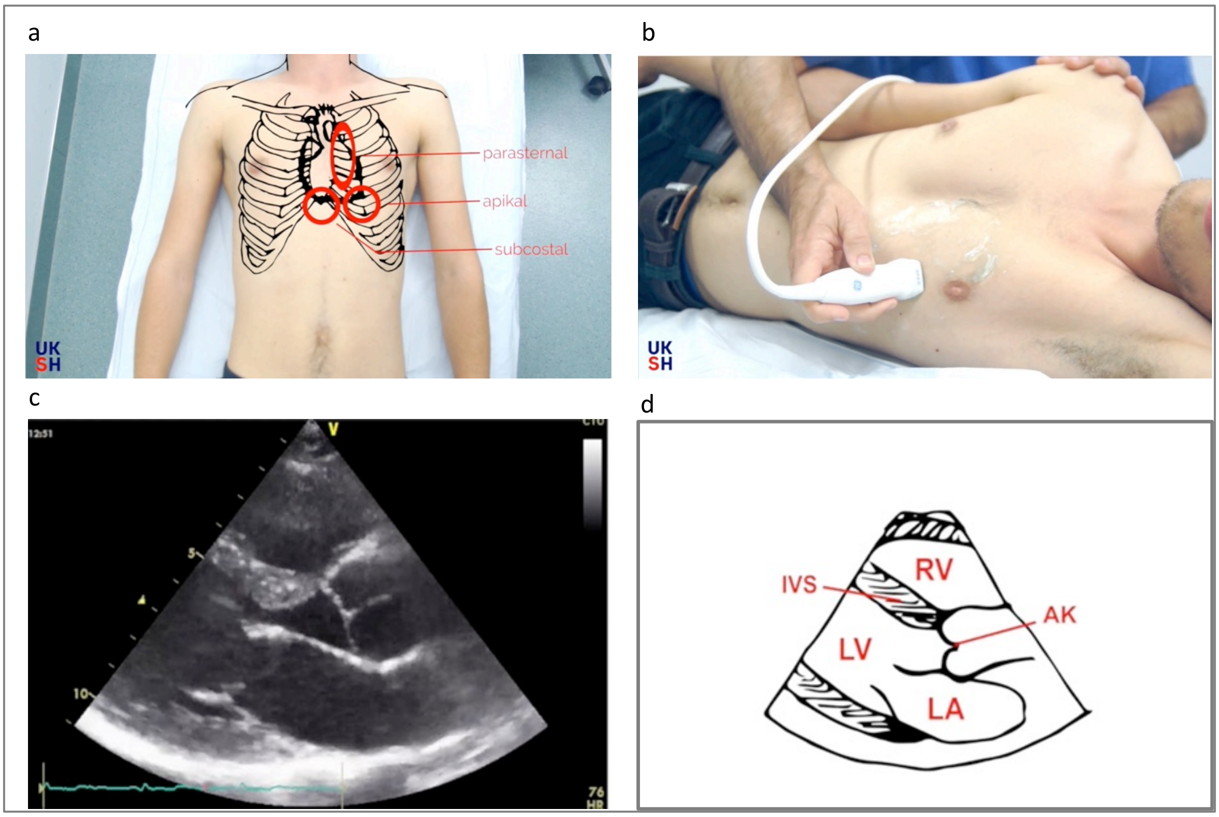

Figure A2. Screen shot example from the TTE video

Panel a shows screen shot for overview thorax anatomy and 3 respective transthoracic echocardiography (TTE) standard views. Panel b shows probe handling and patient positioning for standard view parasternal long-axis. Panel c shows the obtained ultrasound image of the heart from this standard view. Panel d shows the corresponding schematic drawing with the right (RV) and left ventricle (LV), interventricular septum (IVS), aortic valve (AK) und left atrium (LA) as presented in the video.
